# Gene mapping and functional annotation of GWAS of oral ulcers using FUMA software

**DOI:** 10.1038/s41598-020-68976-2

**Published:** 2020-07-22

**Authors:** Xiaoye Jin, Yijie Wang, Xingru Zhang, Wenqing Zhang, Hongdan Wang, Chuanliang Chen

**Affiliations:** 1 College of Forensic Medicine, Xi’an Jiaotong University Health Science Center, Xi’an, China; 2 Key Laboratory of Shaanxi Province for Craniofacial Precision Medicine Research, College of Stomatology, Xi’an Jiaotong University, Xi’an, China; 3 Medical Genetics Institute of Henan Province, Henan Provincial People’s Hospital, Zhengzhou University People’s Hospital, Zhengzhou, China

**Keywords:** Computational biology and bioinformatics, Genetics

## Abstract

Oral ulcers not only influence the physical health of patients, but they also interfere with their quality of life. However, the exact etiology of oral ulcers is not clear. To explore the roles of genetic factors in oral ulcers, a genome-wide association study of the condition in European individuals was re-evaluated by the FUMA v1.3.5e online tool. A total of 380 independent significant single nucleotide polymorphisms (SNPs) and 89 lead SNPs were identified in 34 genomic risk loci. Out of these identified genomic risk loci, 280 possible causal genes were pinpointed by positional mapping and expression quantitative trait locus mapping. Among these genes, 216 novel genes were identified. Furthermore, some genomic loci were mapped to a single gene. Functional annotation of these prioritized genes revealed that the immune response pathway was implicated in the onset of oral ulcers. Overall, our findings revealed novel possible causal genes and demonstrated that the immune response has a crucial role in the occurrence of oral ulcers.

## Introduction

The oral ulcer is an ulcer that occurs on the mucous membrane of the oral cavity. Nearly one-quarter of young adults and many children are affected by this condition^1,2^. Recurrent aphthous stomatitis (RAS) refers to a chronic inflammatory, ulcerative condition of the oral cavity. RAS is characterized by the recurrent outbreak of ulcers and erosions^1^. It is one of the most common causes of oral ulcers, and its prevalence in populations is 5–20%^3^. Even though several factors have been implicated in RAS (e.g., trauma, bacterial/viral infections, anaphylaxis, autoimmune diseases), the exact etiology of this condition is not clear. Dudding and colleagues stated that immune regulation exerts important influences on RAS onset^4^. Besides, family-based studies have revealed that genetic factors also have pivotal roles in RAS etiopathogenesis^5–7^.

Genome-wide association studies can be used to assess associations between SNPs and traits/diseases by detecting a multitude of genetic variants in individuals with different phenotypes. Also, it is beneficial to discern possible causal variants and the genetic architecture of the disease of interest. Dudding et al. re-analyzed data of GWAS of oral ulcers in the UK Biobank Project. They found some genetic variants associated with oral ulcers. Then, they replicated these variants in another novel cohort and assessed the effects of these variants in other populations^4^. Overall, they revealed that 97 variants were related to RAS risk, and that T cell regulation was implicated in RAS^4^. Even though GWAS can provide many disease-associated genetic loci, it may be difficult to determine possible causal mutations because identified genetic variants may be situated in non-coding regions^8^, or be in complete linkage disequilibrium (LD) with unknown causal variants^9^.

Recently, researchers have started to focus on roles of the non-coding regulatory regions in the etiopathogenesis of complex diseases. For example, by assessing mRNA expression data in zebrafish, Golzio et al. searched potential causal genes at genomic risk loci^10^. Wu et al. conducted integration analyses of GWAS results and expression quantitative trait locus (eQTL) data for schizophrenia. They found that some pathways could provide novel insights into the genetic architecture of schizophrenia^11^. Accordingly, integration of GWAS data and gene expression data can be used to identify the potential causal genes associated with complex disease or traits, which are good for carrying out functional experimentation in further research. However, it can be difficult to mine more valuable information from extant GWAS results by integrating functional annotation from Encyclopedia of DNA Elements (ENCODE)^12^, Genotype-Tissue Expression (GTEx) program^13^ or chromatin interaction information^14^. Until now, some tools for bioinformatics analyses have been developed to aid identification of the causal variants associated with traits/diseases^15–17^. Nonetheless, some packages also have some defects when perform post-GWAS analysis. For example, DEPICT and INRICH tools do not take local LD into account, which might induce false-positive enrichment analysis. To provide a highly efficient, concise, and easy-to-use tool, Posthuma et al. developed an Internet-based program named FUMA v1.3.5e (https://fuma.ctglab.nl/) that can further explore GWAS data by utilizing multiple biological databases^18^. Furthermore, FUMA can simultaneously carry out functional annotation of candidate SNPs, gene mapping, tissue-expression analysis of prioritized genes, gene set enrichment analysis (GSEA), and interactive visualization. Posthuma and colleagues re-analysed the GWAS results for Crohn’s disease, schizophrenia and body mass index by FUMA. They found that FUMA not only validated known candidate genes in these traits, it also identified some additional putative causal genes by eQTL mapping and chromatin interaction mapping^18^. Taken together, FUMA could undertake robust and reliable post-GAWS analyses and provide valuable clues for understanding the genetic mechanism of traits/diseases.

We wished to further explore the genetic mechanism of oral ulcers. Previously reported GWAS summary data of oral ulcers^19^ were integrated with the published database by FUMA^18^. First, the most likely causal genes associated with oral ulcers were identified by a combination of positional mapping and eQTL mapping. Next, these prioritized genes were dissected to reveal their molecular function and implicated biological pathways in oral ulcers.

## Methods

### Summary statistics of GWAS for oral ulcers

The GWAS summary data of oral ulcers used in the present study were downloaded from the GWAS ATLAS database^20^. The detailed criteria of sample screening and quality control of SNPs have been presented previously^19^. After quality control of data, 10,599,054 SNPs were used to carry out post-GWAS analyses of oral ulcers. All participants provided their written informed consent. Ethics approval involved in these participants was obtained from the North West Centre for Research Ethics Committee (11/NW/0.382)^19^. All methods used in this study were carried out in accordance with the declaration of Helsinki. The general information of oral ulcers GWAS used in this study was given in Supplementary Table 1.

### Identification of genes and their roles in oral ulcers using FUMA

Definition of genomic risk loci based on oral ulcers GWAS. Independent significant SNPs for which *P* < 5 × 10^−8^ and *r*^2^ < 0.6 were identified from GWAS results. Lead SNPs were defined further from these independent significant SNPs if pairwise SNPs had *r*^2^ < 0.1. Genomic risk loci in which SNPs were in LD (*r*^2^ > 0.6) with independent significant SNPs were identified. The maximum distance between LD blocks to merge into a genomic locus was 250 kb. The genetic data of European populations in 1000G phase3^21^ were viewed as reference data to conduct LD analyses. Besides, 24 SNPs reported by Dudding and colleagues^4^ which reached GWA significant *P*-values and displayed the same effect directions in different independent populations were defined as lead SNPs, as shown in Supplementary Table 2.

Gene mapping. We used two methods to map SNPs to genes. First, CADD scores are deleterious scores of genetic variants obtained by 63 functional annotations. Kircher and colleagues proposed that 12.37 could be viewed as the threshold for a deleterious score^22^. Therefore, SNPs were filtered based on a CADD score > 12.37 when undertaking positional mapping. Then, genes in each genomic risk locus were determined by screened SNPs if the physical distance between a SNP and gene was < 10 kb. Second, for eQTL mapping, SNPs were mapped to a gene if these SNPs had significant effects on expression of the gene. eQTL data of 27 tissues (single-cell RNA eQTL^23^, Database of Immune Cell Expression (DICE)^24^, Biobank-Based Integrative Omics Study (BIOS) QTL browser^25^, and GTEx v8 Whole Blood and Minor Salivary Gland^26^) were used for eQTL mapping. Only significant eQTL values (false discovery rate (FDR) < 0.05) were employed to map SNPs to genes.

Expression (transcripts per million) of prioritized genes in different tissues was estimated from GTEx v8^26^ following winsorization at 50 and log_2_ transformation with pseudocount 1.

GSEA by the GENE2FUN tool in FUMA**.** Using hypergeometric tests, the possible biological functions of the genes identified by positional mapping and eQTL mapping were explored further by comparing them with genes in the GWAS catalog^27^, as well as gene sets in WikiPathways^28^ and the Molecular Signatures Database (MsigDB) v7.1^29^. Overall, there were 20,260 background genes which were applied to GSEA. Gene sets were reported if they met two criteria: (i) at least two prioritized genes belonged to the gene set; (ii) the adjusted *P* value of the gene set was < 0.05. The Benjamini–Hochberg correction^30^ was used to assess the statistical significance of inputted gene sets. Next, genetic correlation analyses between oral ulcers and other available traits/diseases were undertaken by the LD hub^31^.

## Results

### Summary results of GWAS of oral ulcers by FUMA

**Figure 1:**
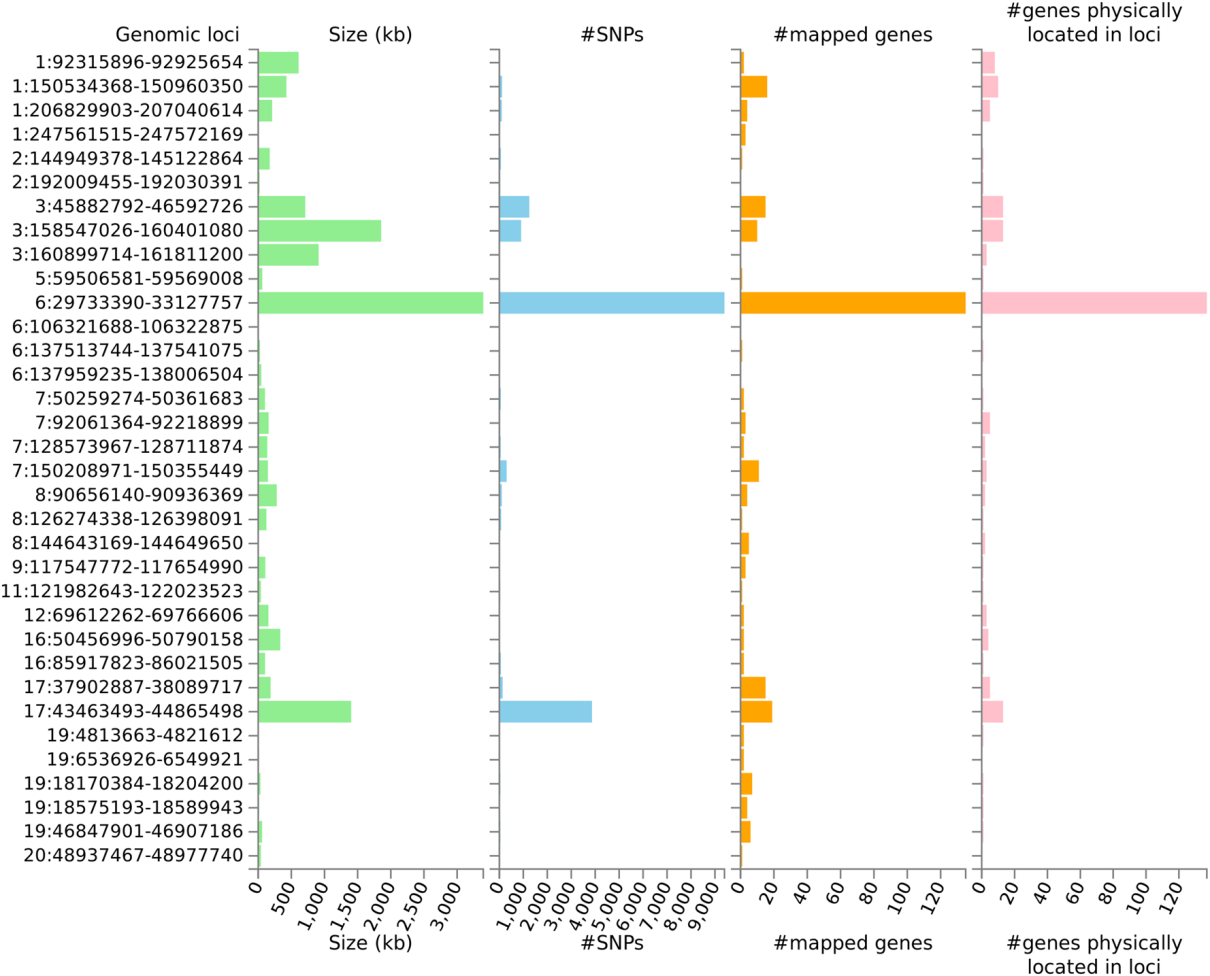
Summary of genomic risk loci based on GWAS of oral ulcers. Genomic risk loci are displayed by the ‘chromosome:start position-end position’ on the Y axis. Histograms from left to right depict the size of the genomic locus, number of candidate SNPs in the genomic locus, number of mapped genes by positional mapping and eQTL mapping in the genomic locus, and the number of genes known to be located within the genomic loci, respectively.

The summary statistics of GWAS for oral ulcers were explored further by FUMA (Table 1). A total of 380 independent significant SNPs and 89 lead SNPs were identified from GWAS of oral ulcers by FUMA (Supplementary Tables 3, 4). For these 89 lead SNPs, we found one stop gained variant, one splice region variant, one non-coding transcript exon variant, three missense variants, five untranslated region variants, 14 regulatory region variants, 24 intergenic variants, and 40 intron variants (Supplementary Table 4). Dudding et al. conducted GWAS of oral ulcers based on the UK Biobank Project, and found 97 independent lead variants^4^. There were 37 lead SNPs which we also found in comparison with the 97 variants discovered by Dudding et al. (Supplementary Table 4). Furthermore, these 89 lead SNPs could be classified into 34 genomic risk loci (Table 2 and Supplementary Table 5). Compared with the 33 risk loci identified in the original study by Bycroft and colleagues^19^, two novel genomic loci (genomic loci 16 and 18) were identified in the present study. We also plotted the results of these 34 genomic risk loci (Fig. 1). Results revealed that most SNPs and genes were mapped in chromosome 6, followed by chromosome 3 and chromosome 17. Furthermore, 280 prioritized genes that may be involved in the biological mechanism of oral ulcers were recognized by FUMA (Supplementary Table 6). Among these genes of interest, 183 genes and 81 genes were located inside and outside genomic risk loci, respectively.

**Table 1:** Summary results of genome-wide analysis of oral ulcers based on FUMA software v1.3.5e.

Index	Number
Independent significant SNPs	380
Lead SNPs	89
Risk loci	34
Positional mapping	143
eQTL mapping	262
Total^#^	280
Genes located outside the risk loci	81
Loci contain prioritized genes	183

^#^The number of unique genes mapped by one of the positional and eQTL mappings.

**Table 2:** Genomic risk loci of interest from oral ulcers GWAS.

Genomic Loci	Start (hg19)	End (hg19)	Rs number	Chromosome	Position (hg19)	*P *value
1	92,315,896	92,925,654	rs141094656	1	92,753,336	2.15E−09
2	150,534,368	150,960,350	rs11204668	1	150,543,686	1.34E−08
3	206,829,903	207,040,614	rs1800871	1	206,946,634	2.57E−66
4	247,561,515	247,572,169	rs72771985	1	247,570,566	4.92E−08
5	144,949,378	145,122,864	rs74966768	2	145,024,540	5.71E−11
6	192,009,455	192,030,391	rs7574070	2	192,010,488	4.91E−19
7	45,882,792	46,592,726	rs4683205	3	46,334,670	8.78E−52
8	158,547,026	160,401,080	rs76830965	3	159,637,678	1.21E−185
9	160,899,714	161,811,200	rs150383292	3	161,279,822	1.31E−11
10	59,506,581	59,569,008	rs9291686	5	59,558,313	1.73E−11
11	29,733,390	33,127,757	rs3135461	6	32,680,122	2.91E−22
12	106,321,688	106,322,875	rs9480610	6	106,321,844	4.42E−08
13	137,513,744	137,541,075	rs4896244	6	137,529,772	1.75E−14
14	137,959,235	138,006,504	rs17264332	6	138,005,515	3.69E−08
15	50,259,274	50,361,683	rs10263046	7	50,309,615	1.21E−18
16	92,061,364	92,218,899	rs112629741	7	92,076,793	2.68E−07
17	128,573,967	128,711,874	rs11761199	7	128,581,835	7.25E−10
18	150,208,971	150,355,449	rs62491812	7	150,327,424	8.14E−07
19	90,656,140	90,936,369	rs11989430	8	90,818,615	8.48E−15
20	126,274,338	126,398,091	rs2385100	8	126,391,143	1.00E−12
21	144,643,169	144,649,650	rs1545536	8	144,643,169	3.92E−09
22	117,547,772	117,654,990	rs10817678	9	117,579,457	1.89E−09
23	121,982,643	122,023,523	rs536991	11	122,008,872	8.26E−09
24	69,612,262	69,766,606	rs1800973	12	69,744,014	3.50E−16
25	50,456,996	50,790,158	rs2066844	16	50,745,926	1.06E−12
26	85,917,823	86,021,505	rs11649485	16	86,014,455	3.85E−18
27	37,902,887	38,089,717	rs12232497	17	38,040,119	4.59E−09
28	43,463,493	44,865,498	rs7210219	17	44,018,519	4.35E−13
29	4,813,663	4,821,612	rs10411970	19	4,814,648	7.03E−12
30	6,536,926	6,549,921	rs28552734	19	6,546,524	1.58E−08
31	18,170,384	18,204,200	rs2305742	19	18,191,441	2.15E−12
32	18,575,193	18,589,943	rs11670056	19	18,589,943	2.45E−09
33	46,847,901	46,907,186	rs3764613	19	46,896,217	2.04E−21
34	48,937,467	48,977,740	rs913678	20	48,955,424	2.69E−14

### Gene prioritization

**Figure 2:**
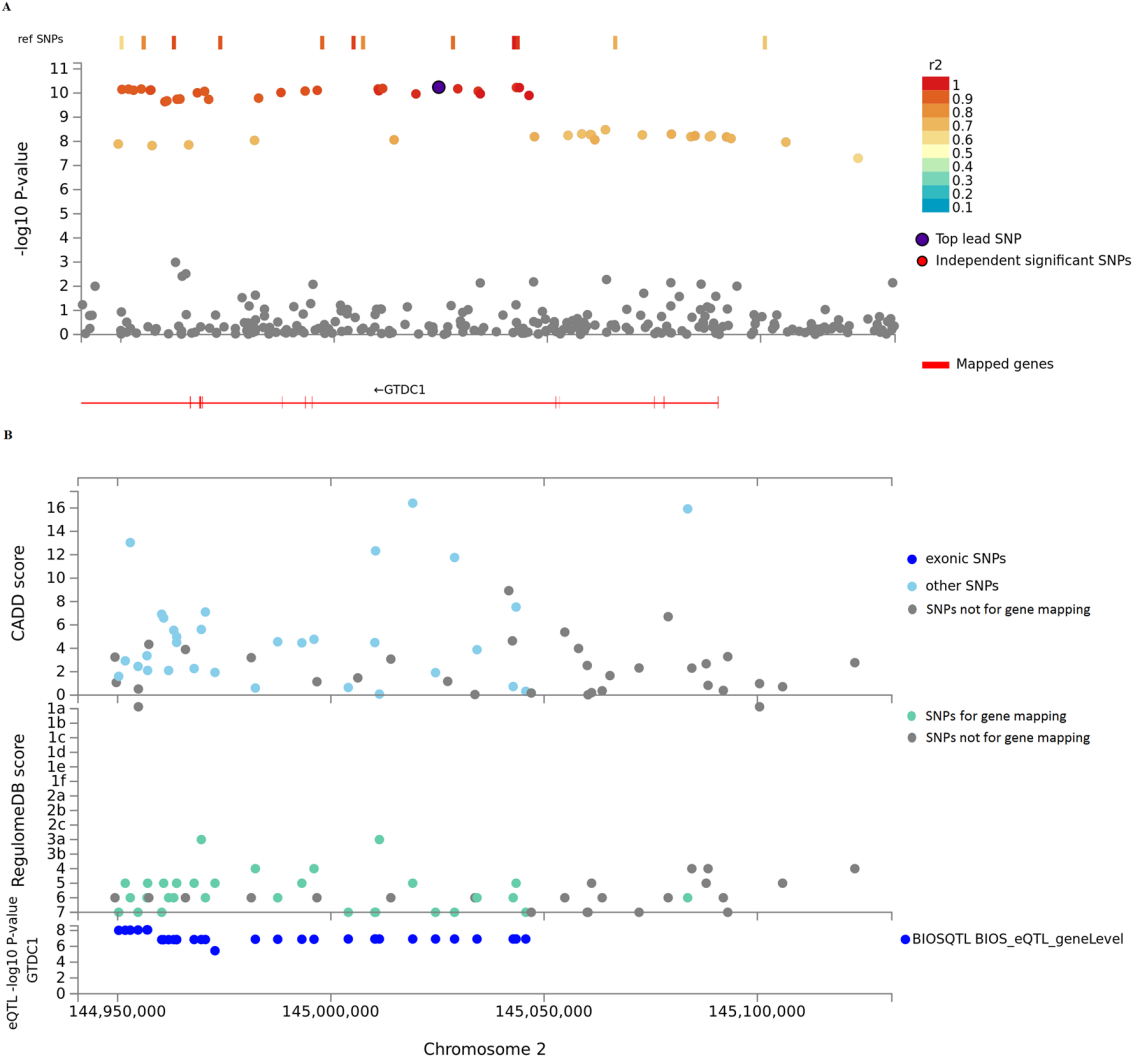
Regional plot of locus 2q22.3 of GWASs of oral ulcers. A, GWAS *P*-values of SNPs in 2q22.3. Genes identified by FUMA v1.3.5e are shown in red. Non-GWAS-tagged SNPs are shown as rectangles because they do not have the *P*-value from GWAS of oral ulcers, but they are in LD with the lead SNP. B, CADD score, RequlomeDB score, and eQTL *P*-value of SNPs in 2q22.3. eQTLs are plotted based on *GTDC1*.

We pinpointed 280 possible causal genes involved in the genetic etiology of oral ulcers by positional mapping and eQTL mapping (Supplementary Table 6). Even though FUMA provides a chromatin-interaction method to map SNPs to genes, chromatin-interaction data related to oral ulcers in FUMA are absent. Therefore, we did not conduct chromatin interaction mapping. For positional mapping, there were 143 genes in 20 genomic risk loci identified by deleterious SNPs. Besides, 262 genes in 28 genomic regions were pinpointed by eQTL mapping. Among these prioritized genes, 125 genes were identified by both deleterious SNPs and eQTL. Out of 34 genomic loci, there were four loci in which no genes were identified; the remaining loci were mapped to at least one candidate gene; nearly half of candidate genes were located in genomic locus 11 (Supplementary Table 6). Compared with findings by Dudding and colleagues^4^, we pinpointed 216 novel genes. Furthermore, we found that six genomic loci were mapped to a single gene (*GTDC1* in genomic locus 5, *NDUFAF2* in genomic locus 10, *IFNGR1* in genomic locus 13, *NSMCE2* in genomic locus 20, *BLID* in genomic locus 23, and *CEBPB* in genomic locus 34), implying that associations between these loci and oral ulcers were likely attributed to these genes. Furthermore, *NDUFAF2*, *CEBPB* and *BLID* were genes identified for the first time in the present study. *NDUFAF2* encodes a complex I assembly factor, which facilitates the translocation of protons from across to inside the mitochondrial membrane. *NDUFAF2* was pinpointed by eQTLs in naïve CD8 T cells, indicating that an SNP may affect *NDUFAF2* expression in CD8 T cells, which further alters the functions of CD8 T cells. *CEBPB*, which was identified by eQTLs in whole blood cells, encodes transcription factors that include a basic leucine zipper domain; *CEBPB* is involved mainly in the regulation of genes related to immune and inflammatory responses. Therefore, abnormal expression of *CEBPB* might lead to dysregulation of the immune and inflammatory response, and then increase the risk of contracting an oral ulcer. *BLID*, which was identified by a deleterious SNP, encodes the BH3-like motif acting on cell death. Therefore, *BLID* may affect the risk of contracting an oral ulcer by regulating cell death. More importantly, we found that *GTDC1* in genomic locus 5 was recognized by both deleterious SNPs and eQTL mapping. Therefore, a regional plot of genomic locus 5 was carried out (Fig. 2). Results revealed that *GTDC1* was prioritized. In the study by Dudding and colleagues, *GTDC1* was also identified by DEPICT software, indicating that *GTDC1* might be implicated in the genetic basis of oral ulcers^4^.

We also estimated the expression patterns of identified genes in different tissues (Supplementary Table 7). Most genes showed consistent expression in these 30 tissues. Moreover, 155 genes showed relatively high expression (> 2.84) in salivary-gland tissues.

### GSEA

GSEA was undertaken to test the possible biological mechanisms of 280 candidate genes implicated in oral ulcers (Supplementary Table 8). A total of 793 gene sets with an adjusted *P* < 0.05 were identified against 20,260 background genes. Among these gene sets, the most significant gene set was the gene set involved in the autism spectrum disorder or schizophrenia (adjusted *P* value = 5.5392E-112), followed by other gene sets involved in other diseases. Furthermore, we found that the pinpointed 280 genes showed strong enrichment signals in gene sets related to the immune response and cytokine regulation, for example: GO_POSITIVE_REGULATION_OF_IMMUNE_RESPONSE (adjusted *P* values = 1.9320E-18), GO_INNATE_IMMUNE_RESPONSE (adjusted *P* values = 6.3002E-18), GO_CYTOKINE_MEDIATED_SIGNALING_PATHWAY (adjusted *P* values = 6.4010E-18), GO_PEPTIDE_ANTIGEN_BINDING (adjusted *P* values = 2.1694E-20), and GO_ANTIGEN_BINDING (adjusted *P* values = 2.7157E-13). Dudding et al. conducted GSEA of identified genes against 14,461 pre-computed gene sets using DEPICT, and found 895 gene sets with a FDR < 0.01^4^. Compared with those 895 gene sets, we identified 693 novel gene sets (Supplementary Table 8). Even so, strong enrichment signals in some T-cell regulatory gene sets were observed in our study and in the study by Dudding and colleagues^4^.

**Figure 3:**
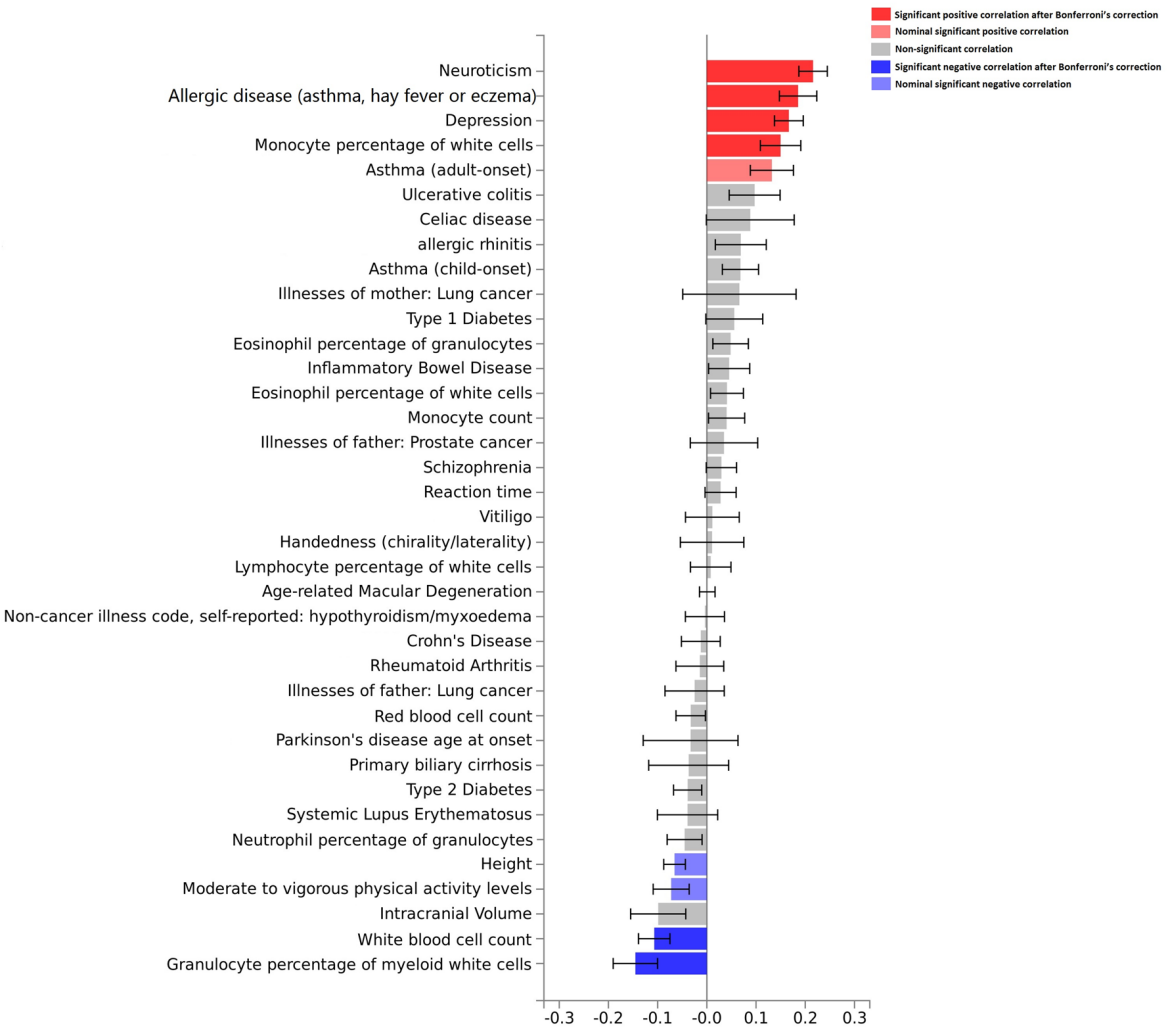
Genetic correlation analyses between oral ulcers and other traits by the LD Hub. Bar chart indicates genetic correlation between two traits and the error bar represents the standard error of the genetic correlation.

To assess further the genetic associations of oral ulcers and other traits, genetic correlation analyses between oral ulcers and these traits were conducted (Fig. 3 and Supplementary Table 9). We found significant positive correlations between oral ulcers and neuroticism, allergic disease (asthma, hay fever or eczema), depression, monocyte percentage of white cells, and asthma (adult onset). We observed significant negative correlations between oral ulcers and height, moderate to vigorous physical activity, white blood cell count, and granulocyte percentage of myeloid cells (*P* < 0.05). However, there were no significant associations between oral ulcers and asthma (adult onset), height or moderate to vigorous physical activity after using the Bonferroni correction.

## Discussion

The factors implicated in RAS are incompletely understood. Possible risk factors are genetic susceptibility, stress, immune-related diseases, as well as a lack of vitamins and minerals^1^. Lake and colleagues assessed the etiology of RAS. They collected incidence information of RAS in twins and their parents. They found that genetic factors contributed to > 60% of variations in RAS onset^32^, implying that genetic factors had pivotal roles in RAS. Using the UK Biobank Project, Bycroft and colleagues collected the phenotypic data of 500,000 individuals and evaluated associations between genetic data and several phenotypes. For RAS, they found that many genetic variations were involved in the risk of suffering from an oral ulcer^19^. In the present study, to further explore the genetic architecture of oral ulcers, post-GWAS analyses of oral ulcers were carried out to pinpoint possible causal variants and genes using FUMA.

Using FUMA, we pinpointed 34 genomic risk loci, including 89 lead SNPs and 380 independent significant SNPs, from GWAS of oral ulcers. Next, these 280 prioritized genes were identified from these 34 genomic risk loci by positional mapping and eQTL mapping. When comparing our results with those of other scholars^4,19^, we not only validated some previous findings, we also obtained novel insights into the genetic architecture of oral ulcers. For instance, 216 out of 280 prioritized genes were not reported in the study by Dudding and colleagues^4^. For these novel identified genes, we found that most genes were located in the human leukocyte region (HLA) region. Genes in the HLA regions might be in strong LD due to complicated LD structure of HLA regions. Thus, most novel genes were found in the HLA region. Moreover, we identified 83 novel genes in other regions. Among these novel prioritized genes, 121 had relatively high expression (> 2.84) in salivary gland tissues, which implied that these genes might have important roles in the onset of oral ulcers. Furthermore, six single genes were found in six genomic regions, respectively. Four genes (*NDUFAF2*, *IFNGR1*, *NSMCE2* and *CEBPB*) showed high expression in most tissues. *GTDC1* showed intermediate expression in these tissues, whereas *BLID* showed low expression in all tissues. Therefore, *BLID* might exert little influence on the onset of oral ulcers given its low expression in these tissues. Further functional validations of these novel genes must be conducted to unveil the effect of these genes in oral ulcers.

GSEA of pinpointed genes revealed that most gene sets implicated in immune-related biological processes had been identified (Supplementary Table 8). Scholars have pointed out that Th1 type immunologic response is closely related to RAS development, and that RAS patients showed higher secretion of Th1 cytokines than that in healthy controls^33–35^. Dudding et al. explored the enrichment patterns of genetic variants related to oral ulcers in regulatory motifs using GARFIELD software. They found that these variants were enriched significantly in DNAsel hypersensitive sites in many T cells, and concluded that identified genes showed tissue-specific expression^4^. We identified GO_T_CELL_ACTIVATION, GO_T_CELL_PROLIFERATION, GO_REGULATION_OF_T_CELL_ACTIVATION and other T cell-related biological processes, implying that T cells have a crucial role in RAS onset. Furthermore, changes in the microbiome community within the oral cavity have also been viewed as risk factors for oral ulcers^36,37^. Dudding and colleagues pointed out that genetic loci associated with oral ulcers might induce oral ulcers by affecting host microbiome compositions; also, the susceptibility of non-infective factors for oral ulcers was influenced by these genetic loci^4^. Therefore, we inferred that genetic variants in genomic risk loci might interfere with the function of immune-related genes, which increases the risk of contracting oral ulcers by eliciting immune response disorders or affecting other risk factors.

Here, we noted genetic correlations between oral ulcers and other traits, especially oral ulcers and neuroticism and depression. Dudding et al. also found significant positive associations between oral ulcers and neuroticism and depression. Furthermore, they conducted local genetic-correlation analyses between oral ulcers and these two traits by the rho-HESS method, and their results revealed that genetic correlations among these traits was scattered evenly in the whole genome^4^. We inferred that there might be a shared genetic architecture among oral ulcers, neuroticism and depression that contributed to these diseases. Besides, the pleiotropy of complex diseases/traits might also lead to their genetic correlations^20^.

In this study, we identified some novel causal genes and gene sets by conducting post-GWAS of oral ulcers based on FUMA. Our data could provide novel insights into the genetic mechanisms of oral ulcers. Nevertheless, our study had three main limitations. First, patients with an oral ulcer were selected by reviewing questionnaire data. This method may have resulted in the misclassification of recruited individuals. Given the short duration of oral ulcers, some affected individuals might not manifest visible symptoms. Therefore, the misclassification of recruited individuals could not be avoided in our study. Besides, RAS and other types of oral ulcers could not be distinguished from each other based on these investigation data. Even so, genetic factors might show slight effects on other types of oral ulcers. More importantly, Dudding and colleagues proposed that the phenomena mentioned-above could elicit small effects on the GWAS of oral ulcers^4^. Second, GWAS of oral ulcers were undertaken in individuals with European ancestries, which revealed the genetic mechanism of the disease in European populations. However, geographic, ethnic, and dietary differences also exert effects on the genetic background of oral ulcers^1^. Consequently, further research on the genetic architecture of oral ulcers in East Asian (especially Chinese) populations should be carried out. Third, the genetic mechanisms of oral ulcers were explored by bioinformatics analysis only. Further validation by cell or tissue experiments for these identified genes was not undertaken. Some genes may show false-positive correlations with oral ulcers if they are located in a LD block which includes causal genes. Taken together, the gene sets presented in our study (especially those for novel genes) require further functional analyses.

## Conclusion

We re-processed the GWAS data of oral ulcers using FUMA, and pinpointed some novel genes associated with oral ulcers. Further functional annotations of prioritized genes revealed that immune regulation pathways were implicated in the risk of contracting oral ulcers. Our results can aid clarification of the genetic architecture of oral ulcers.

## Supplementary information

Supplementary tables.
